# Blood Pressure Mediates the Association Between Visceral-to-Subcutaneous Fat Ratio and Arterial Stiffness in Patients With Type 2 Diabetes

**DOI:** 10.1155/jdr/4898638

**Published:** 2025-10-05

**Authors:** Dou Tang, Xi Gu, Yan Xuan, Fanfan Zhu, Ying Shen, Leiqun Lu

**Affiliations:** Department of Endocrinology, RuiJin Hospital Lu Wan Branch, Shanghai Jiaotong University School of Medicine, Shanghai, China

**Keywords:** baPWV, blood pressure, mediation, Type 2 diabetes mellitus, visceral-to-subcutaneous fat ratio

## Abstract

**Background:**

Emerging evidence links visceral adiposity to arterial stiffness. However, the pathways underlying the association between an elevated visceral-to-subcutaneous fat ratio (V/S ratio) and brachial–ankle pulse wave velocity (baPWV), especially the mediating role of blood pressure, remain unclear in patients with diabetes. We aimed to assess the mediating effects of systolic blood pressure (SBP) and diastolic blood pressure (DBP) on the relationship between the V/S ratio and arterial stiffness in individuals with Type 2 diabetes mellitus (T2DM).

**Methods:**

In this cross-sectional study, 1086 adults with T2DM were enrolled between 2022 and 2024. The visceral fat area (VFA) and subcutaneous fat area (SFA) were assessed using a dual bioelectrical impedance analyzer, and the V/S ratio was calculated as VFA/SFA. Arterial stiffness was evaluated via baPWV. Associations between V/S ratio, blood pressure, and baPWV were examined using multivariable regression. The potential mediating role of blood pressure was further investigated.

**Results:**

After adjusting for confounders, a one-unit increase in the V/S ratio was statistically significantly associated with a 131.81 cm/s higher baPWV (95% CI: 25.67–237.96). The V/S ratio is positively associated with SBP (*β* = 7.76, 95% CI: 1.10–14.42) and DBP (*β* = 3.93, 95% CI: 0.31–7.56). Mediation analysis revealed that SBP and DBP accounted for 41.8% and 33.2% of the effect of the V/S ratio on baPWV, respectively.

**Conclusions:**

An elevated V/S ratio is significantly associated with increased arterial stiffness among patients with T2DM. Moreover, this association may be partially mediated by SBP or DBP.

## 1. Introduction

Cardiovascular disease (CVD) is the leading cause of morbidity and mortality in individuals with Type 2 diabetes mellitus (T2DM), especially in low- and middle-income countries [1, 2]. Individuals with T2DM have more than twice the risk of CVD compared to the general population, highlighting the urgent need for early detection of vascular abnormalities [3, 4]. Arterial stiffness is a significant independent predictor of future cardiovascular events and all-cause mortality [5–7]. Brachial–ankle pulse wave velocity (baPWV), a commonly used noninvasive measure of arterial stiffness, is strongly associated with adverse cardiovascular outcomes in diabetic populations [8].

Obesity, a well-established risk factor for T2DM and CVD, exhibits a complex relationship with arterial stiffening [9–11]. Prior studies have found a positive, negative, or no correlation between body mass index (BMI) and arterial stiffness, as well as waist circumference (WC) [12]. Although general obesity indices such as BMI have been linked to arterial dysfunction, growing evidence suggests that fat distribution, rather than total fat mass, plays a pivotal role in vascular pathology [13]. The visceral-to-subcutaneous fat ratio (V/S ratio), a relative body fat composition metric, has recently been proposed as a better marker of cardiometabolic risk than conventional obesity metrics [14–16]. Notably, Asian populations, including those with T2DM, often exhibit elevated visceral adiposity even at lower BMI levels, highlighting the clinical significance of V/S ratio assessment in this population [17]. However, few studies have examined the relationship between V/S ratio and arterial stiffness in T2DM patients, with conflicting findings reported across studies using traditional obesity measurements [18–20].

Visceral adiposity is a well-recognized risk factor for hypertension, accounting for 65%–78% of primary hypertension [21]. Elevated blood pressure is a major contributor to arterial stiffening [22]. However, it remains unclear whether blood pressure affects the relationship between obesity and arterial stiffness. Therefore, we aimed to determine the association between the V/S ratio and baPWV in patients with T2DM and to evaluate the mediating roles of systolic and diastolic blood pressure (SBP/DBP) in this relationship.

## 2. Methods

### 2.1. Study Design and Patients

We enrolled 1086 consecutive inpatients diagnosed with T2DM at the Department of Endocrinology of RuiJin Hospital Lu Wan Branch, Shanghai Jiaotong University School of Medicine, from March 2020 to December 2024. Patients who underwent measurements of both baPWV and visceral fat area (VFA), as well as subcutaneous fat area (SFA) were included in the cross-sectional study. T2DM diagnosis was established according to the 2020 American Diabetes Association (ADA) criteria [23]. Patients were excluded based on the following criteria: diabetes other than T2DM (*n* = 16), malignancy (*n* = 49), missing data of VFA or SFA (*n* = 21), missing data of baPWV (*n* = 24), or a low ankle–brachial index (< 0.9 in either leg; *n* = 57). For covariates, the proportion of missing data was less than 5% for each variable. Therefore, cases with missing covariate data were also excluded from the corresponding analyses. The number and proportion of missing data for each covariate are summarized in Table S1.

According to the Helsinki Declaration, the study protocol was approved by the Ethics Committee of RuiJin Hospital Lu Wan Branch, Shanghai Jiaotong University School of Medicine. All patients gave their written informed consent.

### 2.2. Clinical and Biochemical Analysis

All patients completed structured interviews by trained research staff, including sex, age, duration of diabetes mellitus, smoking status (never, former, or current), alcohol consumption (never or current), and medical history. BMI was calculated as body weight (kilogram)/height (square meter). SBP and DBP were measured after a 5-min rest period using an electronic sphygmomanometer. Blood samples were collected from patients after an overnight fast of at least 8 h. Fasting blood glucose (FBG), glycated hemoglobin A1c (HbA1c), triglycerides (TG), total cholesterol (TC), high-density lipoprotein cholesterol (HDL-C), low-density lipoprotein cholesterol (LDL-C), uric acid (UA), and renal function tests were measured using standard methods.

### 2.3. Measurements of VFA and SFA

VFA and SFA were assessed using bioelectrical impedance analysis (BIA) with the DUALSCAN HDS-2000 (Omron Healthcare Co., Kyoto, Japan). The fasting patients were placed in a supine position for the examination. The abdominal cross-sectional area was measured at the level of the umbilicus. After placing electrode clips and electrodes on the exposed hands, feet, and abdomen, the abdominal VFA and SFA were measured while the patients held their breath following a quiet exhalation. The V/S ratio was calculated by dividing VFA by SFA [24, 25].

### 2.4. Measurements of baPWV

baPWV was measured using an automatic atherosclerosis detection device (BP-203RPE III, form PWV/ABI, Omron Healthcare Co., Kyoto, Japan). Patients rested in a supine position for at least 5 min before measurement. The arm and ankle cuffs were placed 2–3 cm above the elbow crossbar and 1–2 cm above the inner ankle, respectively. The device automatically calculated the distance between the arm and ankle and measured the time difference between the initial increases in brachial and tibial waveforms. baPWV was calculated by dividing the distance by the time difference. The mean value of left and right baPWV measurements was used for analysis [26].

### 2.5. Statistical Analysis

The characteristics of the patients were illustrated based on V/S ratio tertiles. Continuous variables with normal distributions (assessed by the Kolmogorov–Smirnov tests and *Q*-*Q* plots) were expressed as mean ± standard deviation, while nonnormally distributed variables were reported as median (interquartile range). Categorical variables were presented as frequencies (percentages). One-way ANOVA or the Kruskal–Wallis test was used to compare continuous variables across the three groups. Chi-square test was used for categorical variables. The reported *p* values reflect results from global tests comparing all three groups. Pairwise post hoc comparisons were not performed. The V/S ratio was assessed as a continuous variable and categorized into tertiles, with the lowest tertile serving as the reference group. The associations between V/S ratio and baPWV were assessed using univariate and multivariable linear regression models across three different models. Regression coefficient estimates and 95% confidence intervals (CIs) are reported. The crude model was unadjusted. Model I was adjusted for age and gender. Model II was additionally adjusted for the duration of diabetes, BMI, HbA1c, LDL-C, TG, estimated glomerular filtration rate (eGFR), UA, smoking status, and alcohol consumption. These possible confounders were selected based on their clinical significance, existing scientific literature, or a variation exceeding 10% in effect estimates [27]. The variance inflation factor (VIF) method was used to assess multicollinearity, with a VIF of five or higher indicating multicollinearity.

Mediation analysis was performed to evaluate whether the effect of the exposure variable (V/S ratio) on the outcome variable (baPWV) was mediated by the mediator variable (SBP or DBP), adjusted for age, sex, the duration of diabetes, BMI, HbA1c, LDL-C, TG, eGFR, UA, smoking status, and alcohol consumption. Mediation analysis was performed using the bootstrap method with 1000 resamples to estimate total, direct, and indirect effects and to calculate the proportion mediated (PM). A mediation effect was considered statistically significant if the 95% CI of the *β* coefficient did not include zero [28, 29]. Given the cross-sectional design of this study, causal relationships inferred from the mediation analysis should be interpreted with caution.

Several sensitivity analyses were conducted to test the robustness of our results. First, we excluded patients with V/S ratio values beyond the range of mean ± 3SD and re-estimated the main regression models to assess whether the associations remained consistent. Second, given the sex-related differences in body fat distribution, we conducted sex-stratified analyses. Third, we calculated mean arterial pressure (MAP) using the formula MAP = (SBP + 2∗DBP)/3 and included MAP in both the regression and mediation models to evaluate the joint effect of SBP and DBP. Fourth, we applied generalized additive models (GAMs) with smooth curve fitting to assess the potential linear association between V/S ratio and baPWV. Fifth, we calculated *E*-values to evaluate the potential impact of unmeasured confounders on the conclusions of this observational study.

All analyses were performed using SPSS software (Version 25.0), EmpowerStats, and statistical package R (Version 4.2.0). Mediation analyses were conducted in R using the mediation package. Statistical significance was defined as a two-tailed *p* value < 0.05.

## 3. Results

### 3.1. Patient Characteristics

A total of 1086 patients were enrolled in the study. The V/S ratio levels were distributed in an approximately normal distribution (Figure S1). The baseline characteristics of enrolled patients categorized by tertiles of the V/S ratio are presented in Table 1. Age progressively increased from Tertile 1 to Tertile 3 (*p* = 0.003). In comparison with those in the lowest V/S ratio tertile, patients with elevated levels of V/S ratio had an increasing proportion of males, hypertension, current smoking, and alcohol consumption (all *p* < 0.01). The highest tertile exhibited a lower eGFR and elevated serum UA levels (*p* < 0.01). Additionally, the highest tertile had higher TG median levels and lower HDL-C (*p* < 0.001). BaPWV progressively increased across V/S ratio tertiles, from 1665.34 ± 330.26 cm/s in Tertile 1 to 1737.88 ± 342.14 cm/s in Tertile 3 (*p* = 0.013). BMI, SBP, DBP, FBG, HbA1c, TC, LDL-C, TG, and coronary heart disease incidence across different V/S ratio tertiles did not exhibit significant differences (all *p* values > 0.05).

### 3.2. Univariate Linear Regression of Independent Variables on baPWV

Univariate linear regression showed that age, gender, BMI, V/S ratio, SBP, DBP, the duration of diabetes, FBG, HbA1c, eGFR, dyslipidemia, hypertension, coronary heart disease, and smoking status were associated with baPWV. No significant association was observed between baPWV, UA, HDL-C, and alcohol consumption (Table S2).

### 3.3. Multivariable Regression Analysis of the Association Between V/S Ratio, Blood Pressure, and baPWV

In multivariable regression models (Table 2), the V/S ratio remained significantly associated with baPWV after adjusting for potential confounders in all three models. After adjustment for age, gender, diabetes duration, BMI, HbA1c, LDL-C, TG, eGFR, UA, smoking status, and alcohol consumption, each unit increase in V/S ratio was associated with a 131.81 cm/s elevation in baPWV (95% CI: 25.67–237.96, *p* = 0.015). The significant correlation between the V/S ratio and baPWV remained evident even when the V/S ratio was stratified into tertiles, as shown by the significant trend test results (*p* < 0.05).

Both SBP and DBP showed robust independent associations with baPWV across all models (Table 2). After controlling for potential confounding variables in Model II, each 1 mmHg increase in SBP was associated with a 7.18 cm/s elevation in baPWV (95% CI: 6.31–8.05, *p* < 0.0001), while a 1 mmHg increase in DBP was associated with an 11.28 cm/s increase (95% CI: 9.63–12.93, *p* < 0.0001).

Furthermore, the V/S ratio demonstrated a prominent positive correlation with SBP as well as DBP across all three models (Table 3). Each 1-unit increase in V/S ratio was linked to an increase of 7.76 mmHg in SBP (95% CI: 1.10–14.42, *p* = 0.023) and 3.93 mmHg in DBP (95% CI: 0.31–7.56, *p* = 0.034), even after controlling for all covariates in Model II.

### 3.4. Mediation Analysis

**Figure 1:**
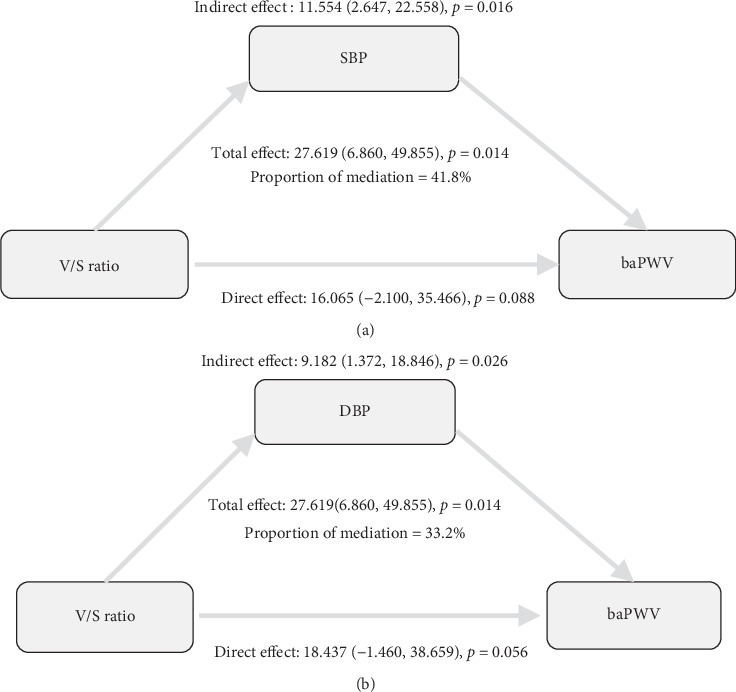
Mediation effect of (a) SBP and (b) DBP in the association between the V/S ratio and baPWV: adjusting for age, sex, the duration of diabetes, BMI, HbA1c, LDL-C, TG, eGFR, uric acid, smoking status, and alcohol consumption. V/S ratio, visceral-to-subcutaneous fat ratio; SBP, systolic blood pressure; DBP, diastolic blood pressure.

As shown in Tables 4 and 5 and Figure 1, a significant indirect effect was observed in all three models (all *p* < 0.05). This suggests that both SBP and DBP partially mediate the association between the V/S ratio and baPWV. After adjusting for all confounders in Model II, the mediation effect of SBP accounted for 41.8% of the association between the V/S ratio and baPWV, while DBP mediated 33.2% of the effect (both *p* < 0.05).

### 3.5. Sensitivity Analysis

To assess the robustness of our findings, several sensitivity analyses were performed. First, after excluding participants with V/S ratio values outside the range of mean ± 3SD, the association between the V/S ratio and baPWV remained robust (Table S3), indicating that the main results were not influenced by extreme values. Second, subgroup analyses by sex showed a significant association in males, whereas in females, the association was positive but not statistically significant; however, there was no significant interaction by sex (*p* for interaction = 0.637), suggesting consistency of this relationship across genders (Table S4). Third, to evaluate the combined effect of SBP and DBP, we examined the mediating role of MAP in the association between the V/S ratio and baPWV. Findings remained consistent, with MAP mediating 44.2% of the association (Tables S5–S7). Additionally, a linear relationship between the V/S ratio and baPWV was observed after adjustment for potential confounders, supporting the use of linear regression models (Figure S2). Lastly, we conducted an *E*-value analysis that yielded a value of 2.89, indicating that the observed association is robust to potential unmeasured confounding.

## 4. Discussion

Our findings indicated a significant association among the V/S ratio, blood pressure, and baPWV in patients with T2DM in China. These associations remained significant even after adjusting for confounding factors. Furthermore, we conducted a mediation analysis, which revealed the important roles of SBP and DBP in linking the V/S ratio with baPWV.

Extensive evidence supports an association between obesity indices and arterial stiffness. However, inconsistent findings across studies may be partially attributed to differences in obesity assessment methods. While BMI remains a widely used clinical metric for obesity, it fails to differentiate lean mass from adipose tissue or capture fat distribution patterns [30]. Emerging studies emphasize the superiority of visceral adiposity indices over BMI in predicting arterial stiffness. Xu et al. investigated 8839 normal-weight Chinese adults with T2DM and revealed that an elevated VFA independently predicts arteriosclerosis, underscoring the critical role of visceral adiposity even in nonobese populations [31]. Similarly, another study of 3758 adults showed that higher VFA, but not BMI or WC, was significantly associated with increased baPWV, even after adjusting for potential confounders [20]. Studies in middle-aged adults have further confirmed that baPWV shows stronger associations with abdominal obesity indicators (e.g., waist-to-hip ratio and visceral fat) compared to general adiposity measures such as BMI, body fat percentage, and SFA [32]. These findings consistently demonstrate that central obesity, particularly visceral fat accumulation, is a stronger determinant of arterial stiffness compared to overall adiposity. This highlights the importance of integrating metrics of fat distribution into cardiovascular risk assessment rather than relying solely on weight-based indices. The V/S ratio, quantifying the balance between visceral adipose tissue and subcutaneous adipose tissue, is a novel obesity metric with unique clinical value in predicting metabolic dysfunction and CVD risk [16, 33, 34]. While CT or MRI is recognized as the gold standard for assessing body fat distribution, previous studies have shown that VFA and SFA measured by dual BIA are comparable in accuracy to those measured by CT [24, 35]. Consistent with previous studies, our findings demonstrate that a higher V/S ratio is strongly associated with increased arterial stiffness in Chinese patients with T2DM.

Obesity, particularly visceral adiposity, is a major contributor to hypertension, accounting for approximately 65%–78% of essential hypertension cases. Mechanisms underlying obesity-related hypertension include kidney compression by perirenal fat, the renin–angiotensin–aldosterone system (RAAS) activation via adipose-derived factors, and sympathetic nervous system stimulation by insulin and leptin [21, 36]. Our findings align with previous studies, confirming a significant positive correlation between the V/S ratio and both SBP and DBP across all models. Additionally, consistent with existing evidence [37, 38], baPWV exhibited a positive association with blood pressure in our research.

To the best of our knowledge, this is the first study to demonstrate that SBP and DBP partially mediate the relationship between the V/S ratio and baPWV in patients with T2DM. This aligns with previous evidence from Liu et al., who used a longitudinal cohort to show that both SBP and DBP predominantly mediate the link between childhood BMI and adult arterial stiffness. Notably, previous research by Liu et al. examined BMI, a measure of general adiposity, in a general population. In contrast, our study focused on the V/S ratio, which indicates visceral fat distribution in a T2DM population. Despite these differences in adiposity metrics and study populations, both studies consistently identified blood pressure as a critical mediator in linking obesity with vascular dysfunction. Sun et al. conducted a cross-sectional study involving 94 heart failure patients with preserved ejection fraction. They found that SBP mediated 53.3% of the relationship between VFA and baPWV, while DBP showed no significant mediation. Although this finding is partially consistent with our results, the discrepancy may be due to differences in study populations and limited sample size. Notably, in our study, the direct effect of the V/S ratio on baPWV became attenuated and nonsignificant in both Model I and Model II, while the indirect effect through SBP and DBP remained statistically significant across all models. The lack of a significant direct effect could be partly attributed to covariate adjustment and sample size limitations.

The observed association may involve interconnected obesity-related pathways. Obesity and insulin resistance trigger activation of the sympathetic nervous system through mechanisms such as adipokine dysregulation, impaired insulin signaling, and chronic inflammation [39, 40]. Proinflammatory adipokines, particularly TNF-*α* and IL-6, directly promote arterial stiffness and resistant hypertension [40]. The RAAS, activated in obesity, hypertension, and diabetes, promotes vascular inflammation and smooth muscle hypertrophy via Angiotensin II signaling and mineralocorticoid receptor–endothelial sodium channel (MR-EnNaC) pathway. Adipokines such as leptin, adiponectin, and resistin further drive vascular remodeling [41]. Insulin resistance exacerbates these processes through SGK-1-mediated endothelial sodium retention and reduced nitric oxide bioavailability [39, 42]. In diabetes, chronic hyperglycemia amplifies vascular stiffening by enhancing oxidative stress and potentiating RAAS activation.

The current study has several limitations. First, due to the cross-sectional nature of our study, definitive causal inferences cannot be made. Although the mediation analysis implies potential directional effects, the lack of temporal data prevents confirmation of the causal sequence and raises the possibility of reverse or bidirectional causation. While our study identified associations among visceral fat, blood pressure, and arterial stiffness, the directionality of these relationships remains uncertain. Visceral fat may contribute to arterial stiffness via blood pressure elevation, but it is also plausible that increased arterial stiffness influences blood pressure or fat distribution. Future longitudinal and mechanistic studies involving molecular and physiological assessments are warranted to clarify the causal pathways linking visceral fat accumulation, hypertension, and vascular remodeling. Second, despite employing multivariable regression models to account for potential confounders, there may still be residual confounding from unmeasured or unknown variables. Third, given that the study population consisted of Chinese patients from a single center, caution should be advised when generalizing these findings to other populations. Fourth, although BIA is a convenient and noninvasive method for assessing body composition, it has certain limitations, including sensitivity to hydration status and device calibration, which may introduce measurement error and reduce its accuracy compared to imaging-based techniques such as CT or MRI.

## 5. Conclusions

In the present study, we demonstrated a significant positive association among the V/S ratio, blood pressure, and baPWV in individuals with T2DM. Notably, both SBP and DBP may partially mediate the relationship between the V/S ratio and arterial stiffness. Our findings suggest that combining adiposity reduction with antihypertensive interventions may synergistically attenuate arterial stiffness progression in T2DM patients.

## Figures and Tables

**Table 1:** Characteristics of patients according to the tertiles of V/S ratio (*n* = 1086).

**Variables**	**Overall**	**Tertile 1** **0.03–0.54** **n** = 362	**Tertile 2** **0.54–0.67** **n** = 362	**Tertile 3** **0.67–1.59** **n** = 362	**p** ** value**
Age (years)	58.81 ± 11.02	57.68 ± 12.34	58.40 ± 10.79	60.36 ± 9.63	0.003
Gender, *n* (%)					
Female	422 (38.86%)	185 (51.10%)	157 (43.37%)	80 (22.10%)	< 0.001
Male	664 (61.14%)	177 (48.90%)	205 (56.63%)	282 (77.90%)	
BMI (kg/m^2^)	25.80 ± 3.75	25.83 ± 4.11	25.91 ± 3.69	25.65 ± 3.42	0.645
VFA (cm^2^)	114.31 ± 45.68	90.04 ± 40.44	114.30 ± 38.19	138.60 ± 44.67	< 0.001
SFA (cm^2^)	189.79 ± 68.50	204.38 ± 80.18	189.69 ± 62.45	175.29 ± 57.85	< 0.001
V/S ratio	0.61 ± 0.18	0.43 ± 0.10	0.60 ± 0.04	0.80 ± 0.13	< 0.001
DBP (mmHg)	74.72 ± 10.86	74.06 ± 10.39	74.81 ± 11.16	75.29 ± 11.01	0.305
SBP (mmHg)	127.38 ± 18.91	126.07 ± 18.00	127.72 ± 19.72	128.35 ± 18.97	0.247
Diabetic duration (years)	9.23 ± 8.52	7.12 (1.25–15.54)	6.71 (1.44–15.23)	7.92 (1.00–15.25)	0.977
FBG (mmol/L)	7.26 ± 2.40	7.17 ± 2.44	7.22 ± 2.25	7.40 ± 2.51	0.389
HbA1c, (%)	8.51 ± 2.11	8.45 ± 2.30	8.40 ± 1.96	8.69 ± 2.04	0.158
eGFR (mL/min/1.73 m^2^)	106.81 ± 27.95	112.43 ± 29.29	105.50 ± 28.30	102.48 ± 25.24	< 0.001
Uric acid (*μ*mol/L)	353.95 ± 89.86	341.42 ± 89.40	357.99 ± 87.97	362.46 ± 91.06	0.004
Triglycerides (mmol/L)	2.17 ± 2.36	1.41 (1.00–2.04)	1.62 (1.20–2.45)	1.75 (1.14–2.49)	< 0.001
Total cholesterol (mmol/L)	5.01 ± 1.36	4.97 ± 1.26	5.06 ± 1.33	5.01 ± 1.49	0.707
HDL-C (mmol/L)	1.15 ± 0.29	1.21 ± 0.34	1.13 ± 0.26	1.10 ± 0.25	< 0.001
LDL-C (mmol/L)	3.21 ± 0.95	3.17 ± 0.92	3.24 ± 0.93	3.22 ± 0.99	0.641
Hypertension, *n* (%)	583 (53.68%)	169 (46.69%)	199 (54.97%)	215 (59.39%)	0.002
Coronary heart disease, *n* (%)	121 (11.14%)	36 (9.94%)	38 (10.50%)	47 (12.98%)	0.384
Smoking status, *n* (%)					< 0.001
Never smoker	600 (55.45%)	232 (64.44%)	214 (59.28%)	154 (42.66%)	
Ex-smoker	120 (11.09%)	32 (8.89%)	33 (9.14%)	55 (15.24%)	
Current smoker	362 (33.46%)	96 (26.67%)	114 (31.58%)	152 (42.11%)	
Alcohol consumption, *n* (%)	109 (10.07%)	29 (8.06%)	29 (8.03%)	51 (14.13%)	0.007
baPWV (cm/s)	1697.36 ± 339.73	1665.34 ± 330.26	1688.85 ± 343.52	1737.88 ± 342.14	0.013

*Note:* Data are presented as mean ± SD, median (interquartile range), or number (%). *p* values represent the results of global tests comparing variables among the three groups.

**Table 2:** Multivariable analysis of the association between V/S ratio and blood pressure with baPWV.

**Variables**	**Crude model**		**Model I**		**Model II**	
	**β** ** (95% CI)**	**p** ** value**	**β** ** (95% CI)**	**p** ** value**	**β** ** (95% CI)**	**p** ** value**
V/S ratio (continuous)	172.45 (58.84, 286.06)	0.003	121.03 (17.94, 224.12)	0.022	131.81 (25.67, 237.96)	0.015
V/S ratio (tertile)						
T1	Reference		Reference		Reference	
T2	23.51 (−25.83, 72.86)	0.350	16.35 (−27.05, 59.74)	0.460	11.75 (−32.49, 56.00)	0.603
T3	72.54 (23.20, 121.89)	0.004	45.95 (0.88, 91.01)	0.046	47.81 (1.35, 94.28)	0.044
*p* for trend	0.004		0.047		0.045	
SBP	7.32 (6.34, 8.30)	< 0.0001	6.81 (5.96, 7.65)	< 0.0001	7.18 (6.31, 8.05)	< 0.0001
DBP	4.76 (2.91, 6.60)	< 0.0001	10.07 (8.46, 11.67)	< 0.0001	11.28 (9.63, 12.93)	< 0.0001

*Note:* Crude model: unadjusted. Model I: adjusted for age and gender. Model II: adjusted for Model I + the duration of diabetes, BMI, HbA1c, LDL-C, TG, eGFR, uric acid, smoking status, and alcohol consumption.

Abbreviations: *β*, regression coefficient; CI, confidence interval; V/S ratio, ratio of visceral to subcutaneous fat area.

**Table 3:** Multivariable regression analysis of V/S ratio and blood pressure.

**Variables**	**Crude model**		**Model I**		**Model II**	
	**β** ** (95% CI)**	**p** ** value**	**β** ** (95% CI)**	**p** ** value**	**β** ** (95% CI)**	**p** ** value**
SBP	8.07 (1.74, 14.41)	0.013	7.55 (1.00, 14.11)	0.024	7.76 (1.10, 14.42)	0.023
DBP	4.17 (0.53, 7.81)	0.025	3.85 (0.26, 7.44)	0.036	3.93 (0.31, 7.56)	0.034

*Note:* Crude model: unadjusted. Model I: adjusted for age and gender. Model II: adjusted for Model I + the duration of diabetes, BMI, HbA1c, LDL-C, TG, eGFR, uric acid, smoking status, and alcohol consumption.

Abbreviations: *β*, regression coefficient; CI, confidence interval.

**Table 4:** Mediation analysis of the association between V/S ratio and baPWV mediated by SBP.

	**Crude model**		**Model I**		**Model II**	
	**β** ** (95% CI)**	**p** ** value**	**β** ** (95% CI)**	**p** ** value**	**β** ** (95% CI)**	**p** ** value**
Total effect	36.394 (14.677, 60.020)	< 0.0001	25.528 (5.159, 48.338)	0.016	27.619 (6.860, 49.855)	0.014
Indirect effect	12.308 (3.076, 22.517)	0.012	10.756 (1.595, 21.119)	0.018	11.554 (2.647, 22.558)	0.016
Direct effect	24.086 (3.780, 45.947)	0.018	14.773 (−3.404, 34.660)	0.114	16.065 (−2.100, 35.466)	0.088
PM, %	33.8%		42.1%		41.8%	
*p* value	0.012		0.030		0.026	

*Note:* Crude model: unadjusted. Model I: adjusted for age and gender. Model II: adjusted for Model I + the duration of diabetes, BMI, HbA1c, LDL-C, TG, eGFR, uric acid, smoking status, and alcohol consumption.

Abbreviations: *β*, regression coefficient; CI, confidence interval; PM, proportion mediated.

**Table 5:** Mediation analysis of the association between V/S ratio and baPWV mediated by DBP.

	**Crude Model**		**Model I**		**Model II**	
	**β** ** (95% CI)**	**p** ** value**	**β** ** (95% CI)**	**p** ** value**	**β** ** (95% CI)**	**p** ** value**
Total effect	36.394 (14.677, 60.020)	< 0.0001	25.528 (5.159, 48.338)	0.016	27.619 (6.860, 49.855)	0.014
Indirect effect	4.023 (0.742, 8.149)	0.020	8.084 (1.224, 15.896)	0.024	9.182 (1.372, 18.846)	0.026
Direct effect	32.371 (9.752, 55.649)	0.002	17.444 (−2.810, 37.878)	0.086	18.437 (−1.460, 38.659)	0.056
PM, %	11.1%		31.7%		33.2%	
*p* value	0.020		0.036		0.036	

*Note:* Crude model: unadjusted. Model I: adjusted for age and gender. Model II: adjusted for Model I + the duration of diabetes, BMI, HbA1c, LDL-C, TG, eGFR, uric acid, smoking status, and alcohol consumption.

Abbreviations: *β*, regression coefficient; CI, confidence interval; PM, proportion mediated.

## Data Availability

The data that support the findings of this study are available from the corresponding author upon reasonable request.

## Supporting Information

Supporting Information Additional supporting information can be found online in the Supporting Information section. Table S1 Percentage of missing values of the covariates. Table S2. The univariate analysis for baPWV. Table S3. Multivariable analysis of the association between V/S ratio and blood pressure with baPWV after excluding outliers beyond ± 3SD. Table S4. Sex-stratified and interaction analyses between V/S ratio and baPWV. Table S5. Association between mean arterial pressure and baPWV in multivariable linear regression models. Table S6. Association between V/S ratio and mean arterial pressure in multivariable linear regression models. Table S7. Mediation analysis of the association between V/S ratio and baPWV mediated by mean arterial pressure.
