# Supplementary material for: Blood Pressure Mediates the Association Between Visceral-to-Subcutaneous Fat Ratio and Arterial Stiffness in Patients With Type 2 Diabetes
Source: J Diabetes Res. 2025 Oct 5;2025:4898638. doi: 10.1155/jdr/4898638 (PMC12515565; doi:10.1155/jdr/4898638)
Supplement: Supporting Information — Additional supporting information can be found online in the Supporting Information section. Table S1 Percentage of missing values of the covariates. Table S2. The univariate analysis for baPWV. Table S3. Multivariable analysis of the association between V/S ratio and blood pressure with baPWV after excluding outliers beyond ± 3SD. Table S4. Sex-stratified and interaction analyses between V/S ratio and baPWV. Table S5. Association between mean arterial pressure and baPWV in multivariable linear regression models. Table S6. Association between V/S ratio and mean arterial pressure in multivariable linear regression models. Table S7. Mediation analysis of the association between V/S ratio and baPWV mediated by mean arterial pressure. [file 4898638.f1.docx]

Supplementary Table 1. Percentage of missing values of the covariates.

| **Covariates** | **Missing, n** | **Missing percentage, %** |
| --- | --- | --- |
| DBP, mmHg | 1 | 0.09% |
| SBP, mmHg | 1 | 0.09% |
| FBG, mmol/L | 9 | 0.83% |
| HbA1c, (%) | 12 | 1.10% |
| eGFR, mL/min/1.73m² | 11 | 1.01% |
| Uric acid, umol/L | 10 | 0.92% |
| Triglycerides, mmol/L | 29 | 2.67% |
| Total Cholesterol, mmol/L | 29 | 2.67% |
| HDL-C, mmol/L | 9 | 0.83% |
| LDL-C, mmol/L | 30 | 2.76% |
| Smoking status, n (%) | 4 | 0.37% |
| Alcohol consumption, n (%) | 4 | 0.37% |

Abbreviations: BMI, body mass index; SBP, systolic blood pressure; DBP, diastolic blood pressure; FBG, fasting blood glucose; HbA1c, glycosylated hemoglobin A1c; eGFR, estimated glomerular; HDL-C, high-density lipoprotein-C; LDL-C, low-density lipoprotein-C.

Supplementary Table 2. The univariate analysis for baPWV

| **Covariate** | **β (95% CI)** | ***P* value** |
| --- | --- | --- |
| Age, years | 14.90 (13.30, 16.51) | <0.0001 |
| Gender |  |  |
| Female | Reference |  |
| Male | -81.26(-122.45, -40.07) | 0.0001 |
| BMI, kg/m^2^ | -15.58 (-20.89, -10.26) | <0.0001 |
| VFA, cm^2^ | -0.45 (-0.90, -0.01) | 0.044 |
| SFA, cm^2^ | -0.71 (-1.00, -0.42) | <0.0001 |
| V/S ratio | 172.45 (58.84, 286.06) | 0.003 |
| SBP, mmHg | 7.32 (6.34, 8.30) | <0.0001 |
| DBP, mmHg | 4.76 (2.91, 6.60) | <0.0001 |
| Diabetic duration, years | 11.34 (9.06, 13.61) | <0.0001 |
| FBG, mmol/L | -8.98 (-17.44, -0.51) | 0.038 |
| HbA1c, (%) | -11.48 (-21.13, -1.83) | 0.0199 |
| eGFR, mL/min/1.73m² | -2.09 (-2.80, -1.37) | <0.0001 |
| Uric acid, umol/L | -0.17 (-0.40, 0.05) | 0.1341 |
| Triglycerides, mmol/L | -17.32 (-25.97, -8.67) | <0.0001 |
| Total Cholesterol, mmol/L | -19.46 (-34.50, -4.41) | 0.0114 |
| HDL-C, mmol/L | 66.12 (-3.80, 136.03) | 0.0641 |
| LDL-C, mmol/L | -24.93 (-46.56, -3.30) | 0.0241 |
| Hypertension |  |  |
| No | Reference |  |
| Yes | 151.43 (111.90, 190.95) | <0.0001 |
| Coronary Heart Disease |  |  |
| No | Reference |  |
| Yes | 76.50 (12.42, 140.59) | 0.0195 |
| Smoke status |  |  |
| Never smoker | Reference |  |
| Ex-smoker | 35.66 (-30.70, 102.01) | 0.2925 |
| Current smoker | -68.26 (-112.42, -24.10) | 0.0025 |
| Alcohol consumption |  |  |
| No | Reference |  |
| Yes | -18.56 (-85.93, 48.81) | 0.5894 |

Note: β, regression coefficient; CI, confidence interval.

Abbreviations: BMI, body mass index; VFA, visceral fat area; SFA, subcutaneous fat area; V/S ratio, visceral-to-subcutaneous fat ratio; SBP, systolic blood pressure; DBP, diastolic blood pressure; FBG, fasting blood glucose; HbA1c, glycosylated hemoglobin A1c; eGFR, estimated glomerular; HDL-C, high-density lipoprotein-C; LDL-C, low-density lipoprotein-C.

Supplementary Table 3. Multivariable analysis of the association between V/S ratio and blood pressure with baPWV after excluding outliers beyond ±3SD.

| **Variables** |  | **Crude Model** | |  | **Model I** | |  | **Model II** | |
| --- | --- | --- | --- | --- | --- | --- | --- | --- | --- |
|  |  | **β (95%CI)** | ***P* value** |  | **β (95%CI)** | ***P* value** |  | **β (95%CI)** | ***P* value** |
| **V/S ratio (continuous)** |  | 184.46 (54.17, 314.75) | 0.006 |  | 121.16 (1.84, 240.47) | 0.047 |  | 128.06 (4.95, 251.16) | 0.042 |
| **V/S ratio (tertile)** |  |  |  |  |  |  |  |  |  |
| T1 |  | Reference |  |  | Reference |  |  | Reference |  |
| T2 |  | 22.81 (-27.08, 72.70) | 0.370 |  | 11.66 (-32.23, 55.56) | 0.603 |  | 5.22 (-39.46, 49.90) | 0.819 |
| T3 |  | 68.11 (18.26, 117.96) | 0.007 |  | 40.84 (-4.66, 86.35) | 0.079 |  | 41.54 (-5.32, 88.41) | 0.083 |
| *P* for trend |  | 0.008 |  |  | 0.080 |  |  | 0.085 |  |

Note: Crude model: unadjusted. Model I: Adjusted for age and gender. Model II: Adjusted for Model I + the duration of diabetes, BMI, HbA1C, LDL-C, TG, eGFR, uric acid, smoking status, and alcohol consumption.

Abbreviations: V/S ratio, ratio of visceral to subcutaneous fat area. β, regression coefficient; CI, confidence interval.

Supplementary Table 4. Sex stratified and interaction analyses between V/S ratio and baPWV.

| **Characteristics** | **No. of Patients** | **β (95%CI)** | ***P* for interaction** |
| --- | --- | --- | --- |
| Gender |  |  | 0.637 |
| Male | 664 | 134.27 (10.89, 257.66) |  |
| Female | 422 | 177.21 (-32.17, 386.60) |  |

Note: adjusted for age, the duration of diabetes, BMI, HbA1C, LDL-C, TG, eGFR, uric acid, smoking status, and alcohol consumption.

Supplementary Table 5. Association between mean arterial pressure and baPWV in multivariable linear regression models

| **Variables** | **Crude Model** | |  | **Model I** | |  | **Model II** | |
| --- | --- | --- | --- | --- | --- | --- | --- | --- |
|  | **β (95%CI)** | ***P* value** |  | **β (95%CI)** | ***P* value** |  | **β (95%CI)** | ***P* value** |
| **MAP** | 8.24 (6.67, 9.82) | <0.0001 |  | 10.34 (9.01, 11.66) | <0.0001 |  | 11.30 (9.95, 12.66) | <0.0001 |

Note: Mean arterial pressure = (SBP + 2*DBP)/3.

Crude model: unadjusted. Model I: Adjusted for age and gender. Model II: Adjusted for Model I + the duration of diabetes, BMI, HbA1C, LDL-C, TG, eGFR, uric acid, smoking status, and alcohol consumption.

Abbreviations: MAP, mean arterial pressure; β, regression coefficient; CI, confidence interval.

Supplementary Table 6. Association between V/S ratio and mean arterial pressure in multivariable linear regression models

| **Variables** | **Crude Model** | |  | **Model I** | |  | **Model II** | |
| --- | --- | --- | --- | --- | --- | --- | --- | --- |
|  | **β (95%CI)** | ***P* value** |  | **β (95%CI)** | ***P* value** |  | **β (95%CI)** | ***P* value** |
| **V/S ratio** | 5.47 (1.35, 9.59) | 0.009 |  | 5.08 (0.87, 9.30) | 0.018 |  | 5.21 (0.95, 9.47) | 0.017 |

Note: Mean arterial pressure = (SBP + 2*DBP)/3.

Crude model: unadjusted. Model I: Adjusted for age and gender. Model II: Adjusted for Model I + the duration of diabetes, BMI, HbA1C, LDL-C, TG, eGFR, uric acid, smoking status, and alcohol consumption.

Abbreviations: V/S ratio, visceral-to-subcutaneous fat ratio; β, regression coefficient; CI, confidence interval.

Supplementary Table 7. Mediation analysis of the association between V/S ratio and baPWV mediated by mean arterial pressure.

|  |  | **Crude Model** | |  | **Model I** | |  | **Model II** | |
| --- | --- | --- | --- | --- | --- | --- | --- | --- | --- |
|  |  | **β (95%CI)** | ***P* value** |  | **β (95%CI)** | ***P* value** |  | **β (95%CI)** | ***P* value** |
| **Total effect** |  | 36.394 (14.677, 60.020) | <0.0001 |  | 25.528 (5.159,48.338) | 0.016 |  | 27.619 (6.860, 49.855) | 0.014 |
| **Indirect effect** |  | 9.328 (2.699, 16.610) | 0.002 |  | 10.988 (2.368, 20.797) | 0.006 |  | 12.206 (2.828, 23.027) | 0.012 |
| **Direct effect** |  | 27.067 (5.891, 48.925) | 0.012 |  | 14.540 (-4.947, 34.387) | 0.124 |  | 15.413 (-2.792, 34.636) | 0.108 |
| **PM, %** |  | 25.6% |  |  | 43.0% |  |  | 44.2% |  |
| ***P* value** |  | 0.002 |  |  | 0.018 |  |  | 0.026 |  |

Note: Crude model: unadjusted. Model I: Adjusted for age and gender. Model II: Adjusted for Model I + the duration of diabetes, BMI, HbA1C, LDL-C, TG, eGFR, uric acid, smoking status, and alcohol consumption.

Abbreviations: MAP, mean arterial pressure; β, regression coefficient; CI, confidence interval; PM, proportion mediated.

**Figure Legends**

**
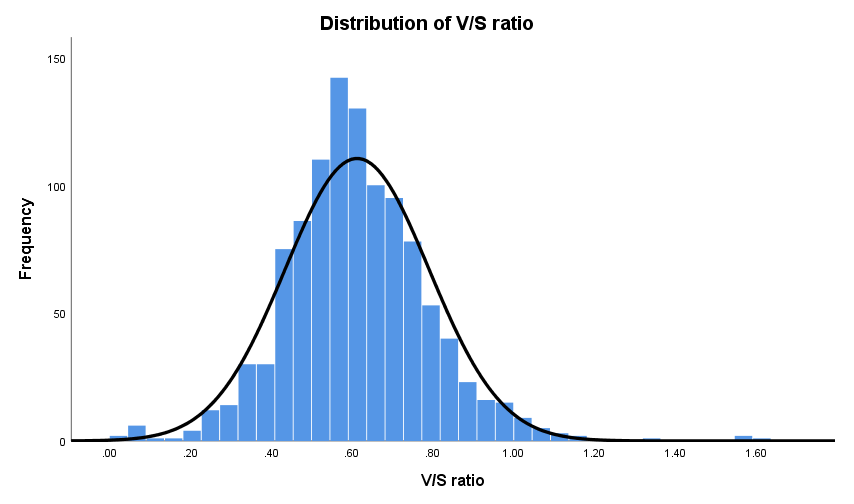
**

Supplementary **Figure 1.** Distribution of V/S ratio. The histogram displays the frequency distribution of V/S ratio values. The black curve represents a normal distribution, indicating that the V/S ratio was approximately normally distributed.


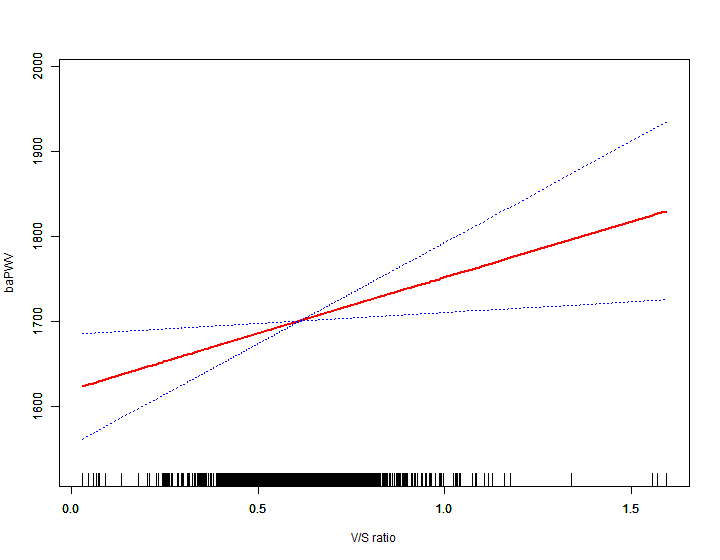


Supplementary **Figure 2.** The linear dose-response relationship between V/S ratio and baPWV. Adjusted for age, sex, the duration of diabetes, BMI, HbA1c, LDL-C, TG, eGFR, uric acid, smoking status, and alcohol consumption. The solid line and dashed line represent the estimated values and their corresponding 95% confidence intervals, respectively.
